# Meteorological determinants of hepatitis E dynamics in Jiangsu Province, China: a pre-COVID-19 era study focusing on multi-route transmission (2005–2018)

**DOI:** 10.3389/fpubh.2025.1604579

**Published:** 2025-08-07

**Authors:** Peihua Li, Jia Rui, Kangguo Li, Deng Bin, Hongjie Wei, Xi Tan, Tianmu Chen

**Affiliations:** ^1^Department of Science and Education, Beijing University of Chinese Medicine Shenzhen Hospital, Shenzhen, China; ^2^State Key Laboratory of Molecular Vaccinology and Molecular Diagnostics, School of Public Health, Xiamen University, Xiamen, China; ^3^Guizhou Center for Disease Control and Prevention, Guiyang, China

**Keywords:** hepatitis E, meteorological determinants, generalized additive model, multi-host and multi-route transmission dynamic model, climate-sensitive dynamics

## Abstract

**Objectives:**

This study aimed to investigate the impact of meteorological factors on the incidence and multi-route transmission dynamics of hepatitis E virus (HEV) in Jiangsu Province, China, during the pre-COVID-19 era (2005–2018), and to develop predictive models for informing public health interventions.

**Study design:**

A dual-model study integrating the Multi-Host and Multi-Route Transmission Dynamic Model (MHMRTDM) and Generalized Additive Model (GAM) was employed to quantify meteorological impacts on multi-route HEV transmission.

**Methods:**

HEV incidence data (2005–2018) and meteorological variables from provincial and national agencies were analyzed. The MHMRTDM quantified transmission rate coefficients (*β*, *β*_w_ and *β*_p_′). GAMs linked the transmission coefficients and incidence to meteorological factors, validated using 2017–2018 data.

**Results:**

The optimal GAM integrated with the MHMRTDM was established (lowest GCV = 1.705 × 10^−21^, *R*^2^ = 0.980, lowest RMSE = 3.682 × 10^−11^, lowest MAE = 2.987 × 10^−11^). Analysis of four dependent variables (incidence, *β*, *β*_w_ and *β*_p_′) revealed distinct climate-driven patterns: (1) Incidence exhibited dual seasonal peaks linked to atmospheric pressure, sunshine duration, and humidity; (2) Host-to-person transmission (*β*_p_′) was most sensitive to climatic conditions, peaking at 1013 hPa and declining sharply above 75% humidity, while susceptible person-to-infected person (*β*) and environment-to-person (*β*_w_) transmission were primarily modulated by humidity and wind speed; (3) The GAM validation confirmed robust performance for transmission coefficients (*p* < 0.001). Predictions for 2019–2021 highlighted persistent seasonal bimodality, reinforcing the model’s utility for outbreak forecasting.

**Conclusion:**

Meteorological factors drive HEV transmission through distinct pathways, with host-to-person interactions being particularly climate-sensitive. While the GAM provided valuable insights, future research incorporating behavioral and land-use factors, as well as causal inference models, will be critical for improving the understanding and predictive accuracy of HEV transmission dynamics.

## 1 Introduction

It is estimated that there are approximately 20 million Hepatitis E virus (HEV) infections worldwide annually, resulting in about 3.3 million symptomatic cases of hepatitis E. According to the World Health Organization, hepatitis E caused approximately 44,000 deaths in 2015, accounting for 3.3% of viral hepatitis-related mortality. China, located in the eastern region of Asia, is an area where hepatitis E is highly endemic.

HEV transmission, primarily occurring via the fecal-oral route through contaminated water and food, is climate-sensitive, with meteorological factors playing a significant role (1–3). While sporadic cases in developed countries arise from zoonotic foodborne transmission (undercooked meat/offal) (4–7), developing countries face waterborne epidemics where seasonal rainfall/monsoons (8, 9) intensify sewage contamination and drought elevates HEV concentrations in domestic water (10, 11). Poor sanitation, limited access to safe water, and insufficient health education further amplify infection risks (12). However, in developed countries, recent travel to endemic regions and consumption of unsafe water have emerged as significant factors contributing to infection (13).

Since the early 20th century, compartmental models have been a cornerstone in infectious disease modeling. Transmission dynamics models can simulate the spread of viral hepatitis by incorporating its transmission characteristics (14–16). Current research models for hepatitis E primarily rely on experimental animal models and basic statistical models. In our previous studies, we developed a Multi-Host and Multi-Route Transmission Dynamic Model (MHMRTDM) to explore the transmission characteristics of HEV (17, 18), which effectively fitted incidence data and analyzed the impact of various intervention measures in the region. Additionally, many research teams have employed spatial autocorrelation analysis or spatial regression analysis to investigate the influence of climate on hepatitis E (19–22). Other studies have designed methods such as Spearman correlation or ensemble learning (23, 24). Especially, generalized additive models (GAMs) have become increasingly pivotal in infectious disease modeling, particularly for characterizing nonlinear relationships between environmental drivers and transmission dynamics (25, 26). However, no studies have yet developed a Generalized Additive Model (GAM) to investigate the impact of meteorological factors on hepatitis E.

Therefore, this study employs a GAM to explore the relationship between meteorological conditions and the incidence and transmission of HEV in Jiangsu Province from 2005 to 2018. The aim is to identify an appropriate model for quantifying meteorological impacts on multi-route HEV transmission and to provide a scientific basis for intervention strategies.

## 2 Materials and methods

### 2.1 Study area

Jiangsu Province is located in the eastern coastal region of mainland China, spanning latitudes 30°45′ to 35°08′N and longitudes 116°21′ to 121°56′E. The province experiences a transitional climate from temperate to subtropical zones, characterized by mild temperatures, moderate precipitation, and distinct seasons. The Huai River and the Northern Jiangsu Irrigation Canal serve as a boundary, dividing the province into two climatic zones: the northern region features a warm temperate humid and semi-humid monsoon climate, while the southern region exhibits a subtropical humid monsoon climate.

### 2.2 Data collection

The incidence data of hepatitis E in Jiangsu Province from 2005 to 2018 were provided by the Jiangsu Provincial Center for Disease Control and Prevention (CDC). This pre-COVID-19 dataset ensures that our analysis reflects baseline transmission patterns independent of pandemic-related disruptions. Meteorological data from January 2005 to March 2017 were collected from the Jiangsu Provincial Meteorological Bureau, including average atmospheric pressure (1 hPa), precipitation (1 mm), relative humidity (1%), sunshine duration (1 h), average temperature (1°C), and average wind speed (1 m/s). These data were aggregated on a monthly basis for fitting the GAM.

From April 2017 to December 2021, meteorological data were obtained from the China Meteorological Administration, including average atmospheric pressure (1 hPa), precipitation (1 mm), relative humidity (1%), sunshine duration (1 h), average temperature (1°C), and average wind speed (1 m/s). To ensure consistency with the monthly time step used in both the MHMRTDM and GAM models, the meteorological data for the period, which were initially recorded on a daily basis, were aggregated to monthly averages. These monthly-averaged meteorological data were then used for the validation and prediction of the model. This adjustment ensures compatibility between the model’s temporal resolution and the input data, improving the robustness of the validation process.

### 2.3 Multi-host and multi-route transmission dynamic model (MHMRTDM)

This study utilized the established MHMRTDM for hepatitis E to calculate the transmission rate coefficients in Jiangsu Province from 2005 to 2018 (18). These coefficients included the susceptible person-to-infected person contact rate coefficient (*β*), the reservoir-to-person contact rate coefficient (*β*_w_), and the host-to-person contact rate coefficient (*β*_p_′). Individual transmission rate coefficients were used to represent different transmission pathways of hepatitis E (Supplementary Figure S1), and their temporal trends were analyzed to reflect the dynamic infection process.

### 2.4 Study design

**Figure 1:**
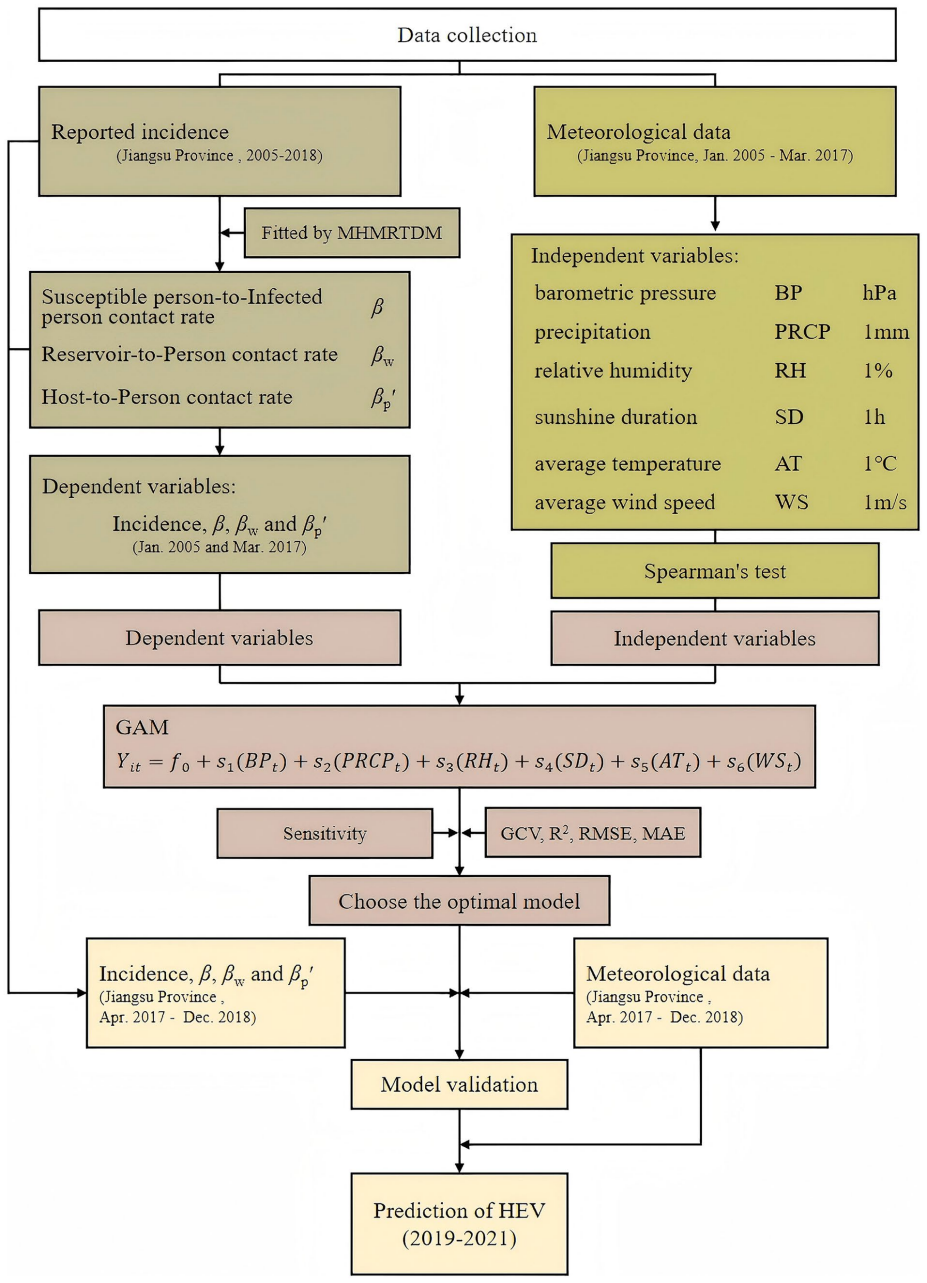
Study design of meteorological factors affect the HEV transmission.

The MHMRTDM was used to calculate the transmission rate coefficients (*β*, *β*_w_, and *β*_p_′) for hepatitis E transmission across various routes (person-to-person, reservoir-to-person, and host-to-person). These transmission rate coefficients and reported incidence were then treated as dependent variables in the GAM, which was used to quantify the relationship between meteorological factors and the transmission dynamics of hepatitis E. The study design is illustrated in Figure 1.

### 2.5 Construction of the generalized additive model (GAM)

Previous studies have demonstrated that the relationship between meteorological factors and infectious diseases is complex. Therefore, this study employed GAM, using the raw incidence data, *β*, *β*_w_, and *β*_p_′ as dependent variables, and average atmospheric pressure (BP, hPa), precipitation (PRCP, mm), relative humidity (RH, %), sunshine duration (SD, h), average temperature (AT, °C), and average wind speed (WS, m/s) as independent variables. The complete model is as follows:


$$\begin{array}{l} Y_{\mathrm{it}}=f_{0}+s_{1}({\mathrm{BP}}_{t})+s_{2}({\text{PRCP}}_{t})+s_{3}({\mathrm{RH}}_{t})+\\ s_{4}({\mathrm{SD}}_{t})+s_{5}({\mathrm{AT}}_{t})+s_{6}({\mathrm{WS}}_{t}) \end{array}$$

Here, *Y_it_* represents the value of different dependent variables (reported incidence, *β*, *β*_w_, or *β*_p_′) on day *t*, and *t* denotes the time point of the independent variables. The GAM was employed to model the non-linear relationship between the meteorological factors and the transmission rate coefficients (*β*, *β*_w_, and *β*_p_′), as well as the incidence of hepatitis E. The smoothing function effectively dealt with the non-linearity of the relationships between independent and dependent variables.

We standardized the *β* parameters to z-scores and assessed their sensitivity to six climate variables (temperature, precipitation, humidity, pressure, wind speed, sunshine hours) through Pearson correlation, linear regression slopes, and generalized additive models (GAMs) (Supplementary Table S1). Sensitivity was visualized using scatter plots with GAM-fitted smooth curves, arranged in a 2×3 grid (Supplementary Figure S2).

### 2.6 GAM validation and prediction

The GAM model was validated using incidence data, the transmission rate coefficients and corresponding meteorological data from April 2017 to December 2018 in Jiangsu Province to prevent overfitting. Subsequently, the established and validated GAM was used to predict the incidence and the transmission rate coefficients of hepatitis E in Jiangsu Province from 2019 to 2021.

### 2.7 Data analysis

Berkeley Madonna 8.3.18 was used to calculate transmission rate coefficients and conduct intervention simulations. Spearman’s correlation test was employed to assess correlations among independent variables, with caution exercised for correlation coefficients greater than 0.7. The “MGCV” package in R 3.2.3 was utilized to construct the GAM and perform predictions. The GAM was estimated using maximum likelihood, and different GAMs were selected based on *R*^2^, generalized cross-validation (GCV) score, root mean square error (RMSE) score and mean absolute error (MAE) score. Figures were generated using the “ggplot2” package in R 3.2.3, and tables were created using Excel 2019.

## 3 Results

### 3.1 Descriptive characteristics of the data

**Figure 2:**
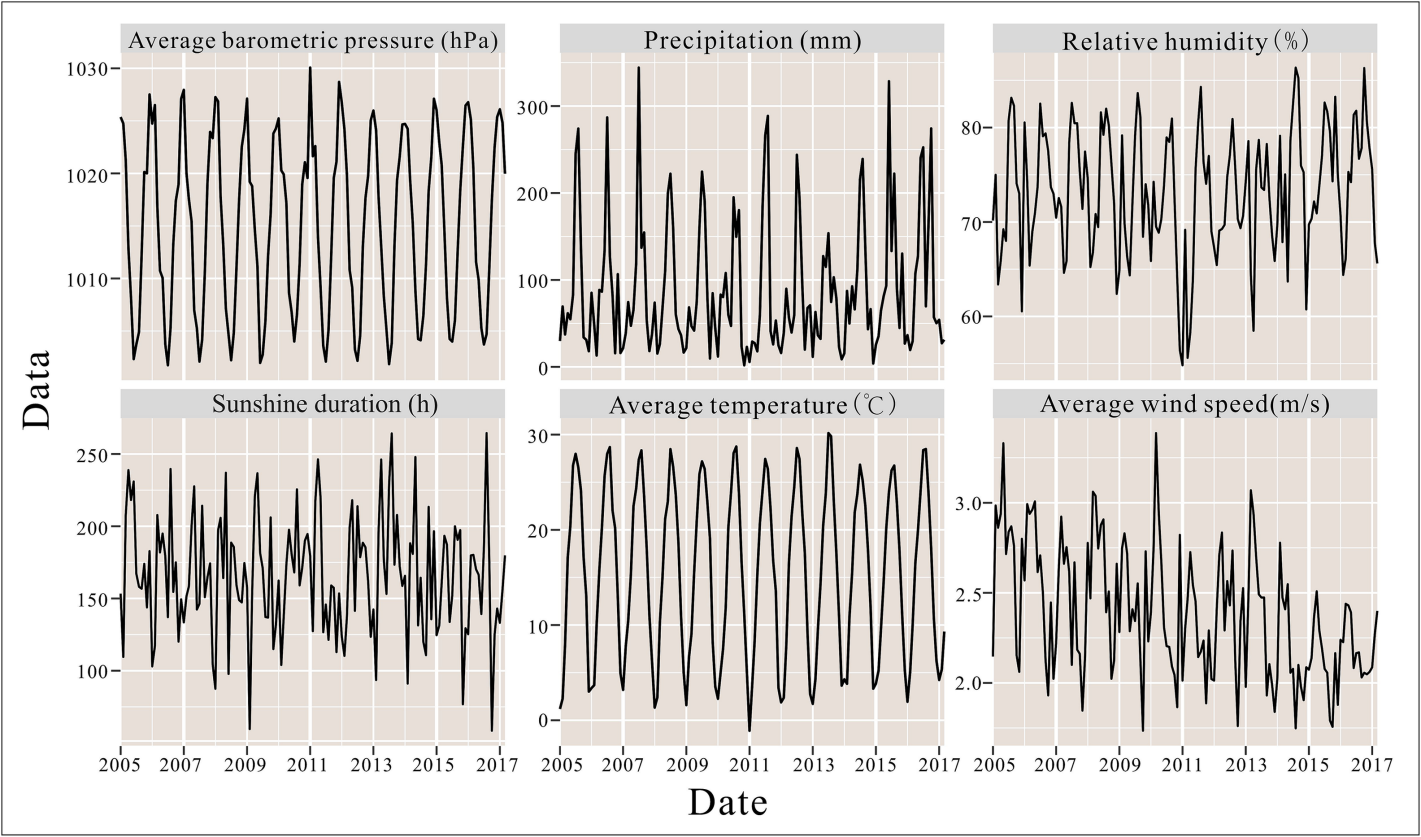
Seasonal patterns in the distribution of meteorological factors.

From 2005 to 2018, a total of 44,923 hepatitis E cases were reported in Jiangsu Province, China, with an average annual incidence rate of 4.12 per 100,000 population. The annual incidence of hepatitis E in Jiangsu Province peaked in 2007 (4.35 per 100,000), 2011 (5.21 per 100,000), and 2013 (4.94 per 100,000), while the lowest incidence was recorded in 2005 at 2.96 per 100,000 (Figure 2). Seasonal patterns in the distribution of meteorological factors over time are also evident in Figure 2, with line graphs for average atmospheric pressure, precipitation, and temperature showing distinct seasonal trends.

During the study period, the highest average temperature in Jiangsu Province was 30.16°C, with average atmospheric pressure ranging from 1001.74 to 1030.07 hPa, sunshine duration from 58.50 to 264.48 h, relative humidity from 54.83 to 86.35%, precipitation from 1.84 to 344.22 mm, and wind speed from 1.73 to 3.39 m/s. Detailed summaries of meteorological factors in Jiangsu Province are presented in Table 1.

**Table 1:** Description of meteorological factors in Jiangsu Province, China.

Meteorological factors	Average (SD)	Minimum	Maximum	P_25_	Median	P_75_
Average barometric pressure (hPa)	1015.270 (8.302)	1001.738	1030.070	1006.985	1017.170	1022.583
Sunshine duration (h)	165.997 (40.715)	58.496	264.483	137.157	164.462	193.391
Precipitation (mm)	89.190 (76.323)	1.843	344.215	32.174	66.148	126.977
Average temperature (°C)	15.498 (9.025)	−1.096	30.161	6.674	16.630	23.778
Relative humidity (%)	73.185 (6.692)	54.826	86.348	69.000	73.826	78.652
Average wind speed (m/s)	2.391 (0.360)	1.735	3.387	2.091	2.365	2.708

### 3.2 MHMRTDM fitting results

**Figure 3:**
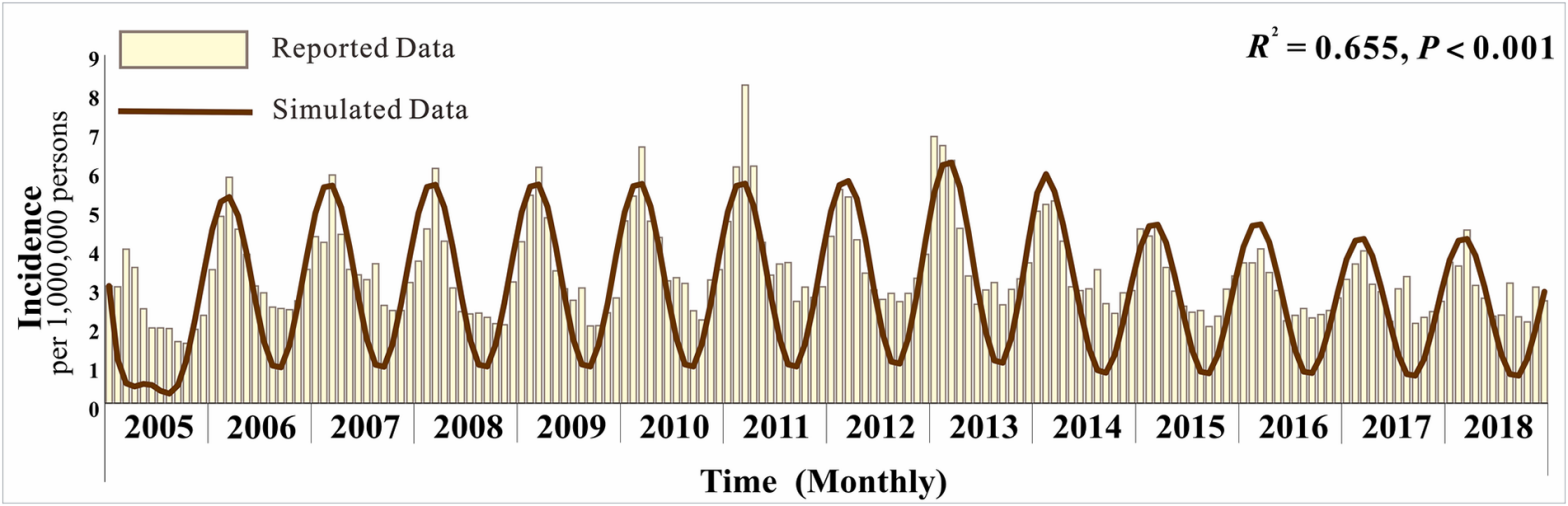
Simulated situation of hepatitis E incidence in Jiangsu Province.

Figure 3 displays the monthly actual incidence rates and the fitted incidence rates, revealing that hepatitis E in Jiangsu Province exhibits seasonal variations with an initial upward trend followed by a decline. Overall, the simulated monthly reported cases align well with the actual trends (*R*^2^ = 0.655; *p* < 0.001).

### 3.3 Correlation analysis of meteorological factors

**Figure 4:**
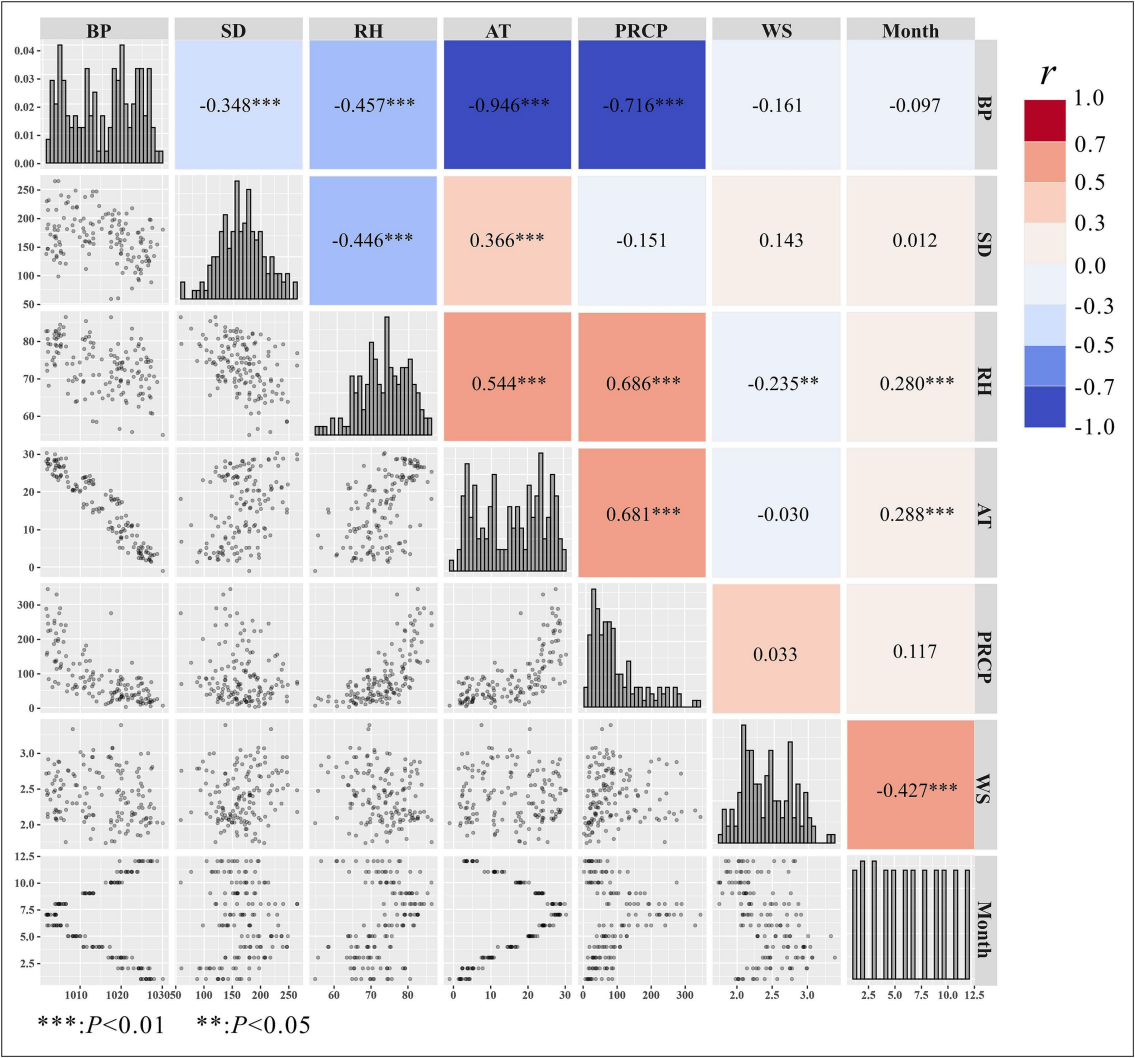
Spearman’s correlation analysis of meteorological factors. BP, barometric pressure; PRCP, precipitation; RH, relative humidity; SD, sunshine duration; AT, average temperature; WS, average wind speed.

Based on Spearman’s correlation analysis of meteorological factors, we found that atmospheric pressure was negatively correlated with other factors, showing a strong correlation with temperature (*r* = −0.946). Additionally, sunshine duration was positively correlated with temperature but negatively correlated with atmospheric pressure and relative humidity. Relative humidity was positively correlated with temperature, precipitation, and month, but negatively correlated with atmospheric pressure and sunshine duration. Temperature showed no correlation with wind speed but was positively correlated with precipitation and relative humidity. Precipitation was not correlated with sunshine duration but was positively correlated with relative humidity and temperature. Wind speed was positively correlated with month but negatively correlated with relative humidity, as shown in Figure 4. Spearman’s correlation analysis revealed that average atmospheric pressure was highly correlated with average temperature (*r* = −0.946, *p* < 0.01) and precipitation (*r* = −0.716, *p* < 0.01). Therefore, atmospheric pressure was not included simultaneously with these two factors in the same GAM during modeling.

### 3.4 Construction of the GAM model

The GAM was constructed using average temperature, relative humidity, sunshine duration, precipitation, month, and other variables as predictors. Based on the generalized cross-validation (GCV) score and *R*^2^, the optimal GAM for each dependent variable with the lowest GCV score is presented in Table 2. The results in Table 2 indicate that the most suitable dependent variable for meteorological factors was the susceptible person-to-infected person contact rate coefficient, which had the lowest GCV score (GCV = 1.705 × 10^−21^, *R*^2^ = 0.980, RMSE = 3.682 × 10^−11^, MAE = 2.987 × 10^−11^).

**Table 2:** Optimal generalized additive models and performance metrics.

Dependent variables	Formula	*R* ^2^	GCV	RMSE	MAE
Reported incidence rates	*f*_0_ + s_1_(BP_t_) + s_2_(SD_t_) + s_3_(RH_t_) + s_4_(Month_t_) + s_5_(WS_t_)	0.704	5.225 × 10^−13^	6.472 × 10^−7^	4.698 × 10^−7^
*β*	*f*_0_ + s_1_(SD_t_) + s_2_(RH_t_) + s_3_(AT_t_) + s_4_(PRCP) + s_5_ (Month_t_) + s_6_(WS_t_)	0.968	1.732 × 10^−10^	1.176 × 10^−5^	8.215 × 10^−6^
*β*_w_	*f*_0_ + b_1_SD_t_ + s_1_(RH_t_) + s_2_(AT_t_) + s_3_(PRCP) + s_4_ (Month_t_) + b_2_WS_t_	0.973	1.781 × 10^−16^	1.196 × 10^−8^	8.915 × 10^−9^
*β_p_*’	*f*_0_ + s_1_(BP_t_) + b_1_SD_t_ + s_2_(RH_t_) + s_3_ (Month_t_) + b_2_WS_t_	0.980	1.705 × 10^−21^	3.682 × 10^−11^	2.987 × 10^−11^

### 3.5 Relationship between incidence, transmission rate coefficients, and meteorological factors

**Figure 5:**
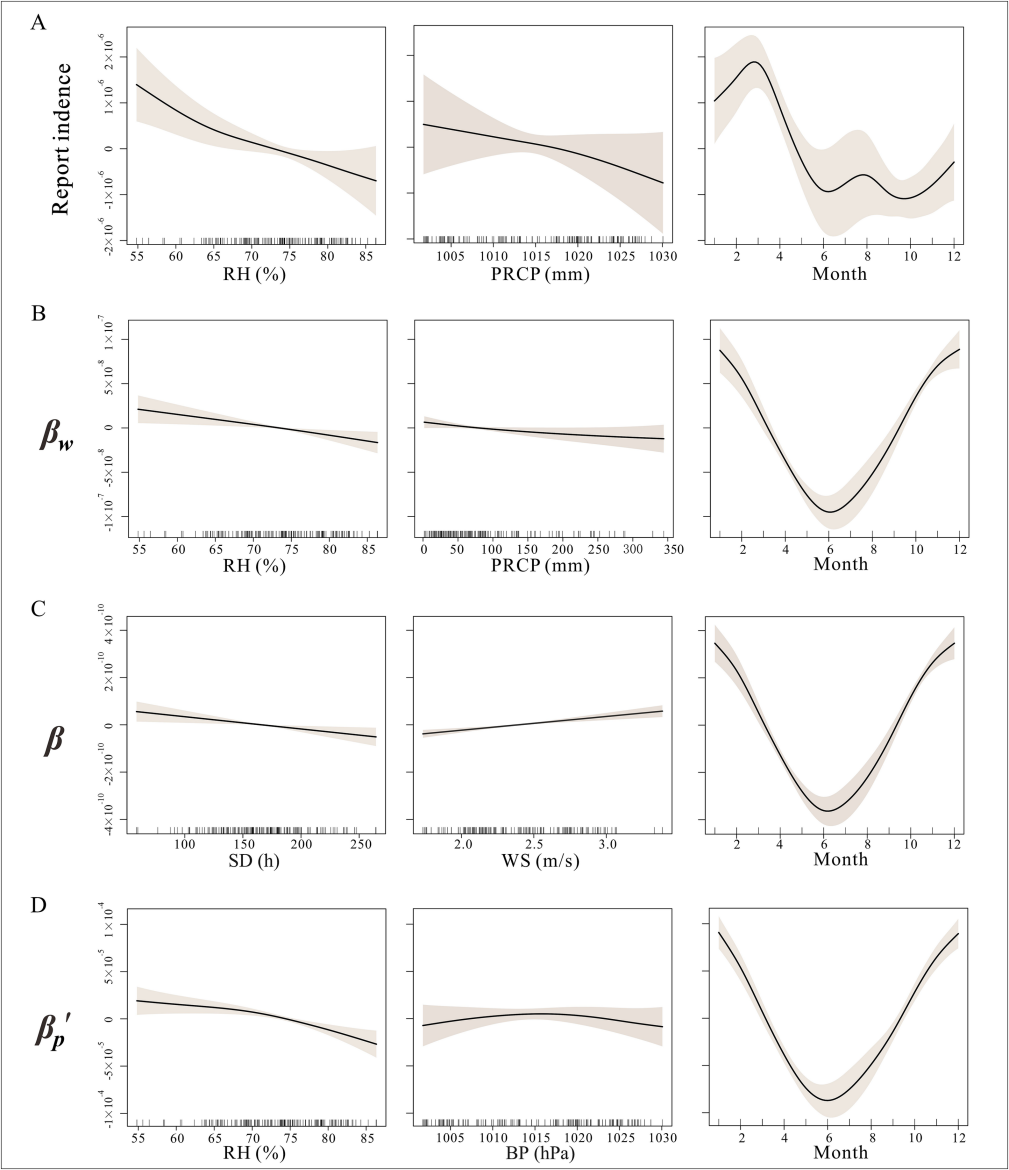
Non-linear relationship between incidence and different transmission with meteorological factors in Jiangsu Province BP, barometric pressure; PRCP, precipitation; RH, relative humidity; SD, sunshine duration; AT, average temperature; WS, average wind speed. **(A)** Relationship between incidence and meteorological factors; **(B)** Relationship between reservoir-to-person transmission and meteorological factors; **(C)** Relationship between susceptible person-to-infected person transmission and meteorological factors; **(D)** Relationship between host-to-person transmission and meteorological factors.

In the GAM for reported incidence rates, all included factors exhibited nonlinear relationships (Figure 5).

#### 3.5.1 Relationship between incidence and meteorological factors

As shown in Figure 5A, the relationships between incidence and average atmospheric pressure, sunshine duration, and relative humidity were nonlinear. Specifically, these three meteorological factors showed a trend where incidence decreased as their values increased. In terms of monthly patterns, hepatitis E incidence exhibited dual peaks in March and August.

#### 3.5.2 Relationship between susceptible person-to-infected person transmission and meteorological factors

As illustrated in Figure 5C, for the susceptible person-to-infected person contact rate coefficient, the GAM simulated the effects of sunshine duration, relative humidity, temperature, and precipitation as nonlinear. The effects of temperature and precipitation were not significant (*p* > 0.05). Relative humidity and sunshine duration showed a trend where the transmission rate decreased as their values increased, while the transmission rate coefficient increased with higher wind speeds.

#### 3.5.3 Relationship between reservoir-to-person transmission and meteorological factors

For the reservoir-to-person contact rate coefficient, all factors except sunshine duration exhibited nonlinear effects, with average temperature and precipitation showing no significant impact (*p* > 0.05). The transmission rate coefficient decreased with increasing relative humidity, temperature, and precipitation. The monthly pattern for reservoir-to-person transmission was consistent with susceptible person-to-infected person transmission, with only a single trough in June (Figure 5B).

#### 3.5.4 Relationship between host-to-person transmission and meteorological factors

In the GAM for the host-to-person contact rate coefficient, sunshine duration and wind speed had linear effects, while atmospheric pressure and relative humidity exhibited nonlinear effects. Relative humidity showed a trend where host-to-human transmission initially decreased and then increased with higher humidity. Atmospheric pressure demonstrated a trend where host-to-person transmission initially increased and then decreased with rising pressure, with the strongest transmission occurring between 1,013 and 1,017 hPa. The monthly pattern for reservoir-to-person transmission was consistent with susceptible person-to-infected person transmission, with only a single trough in June (Figure 5C).

### 3.6 Validation and prediction of the GAM model

**Figure 6:**
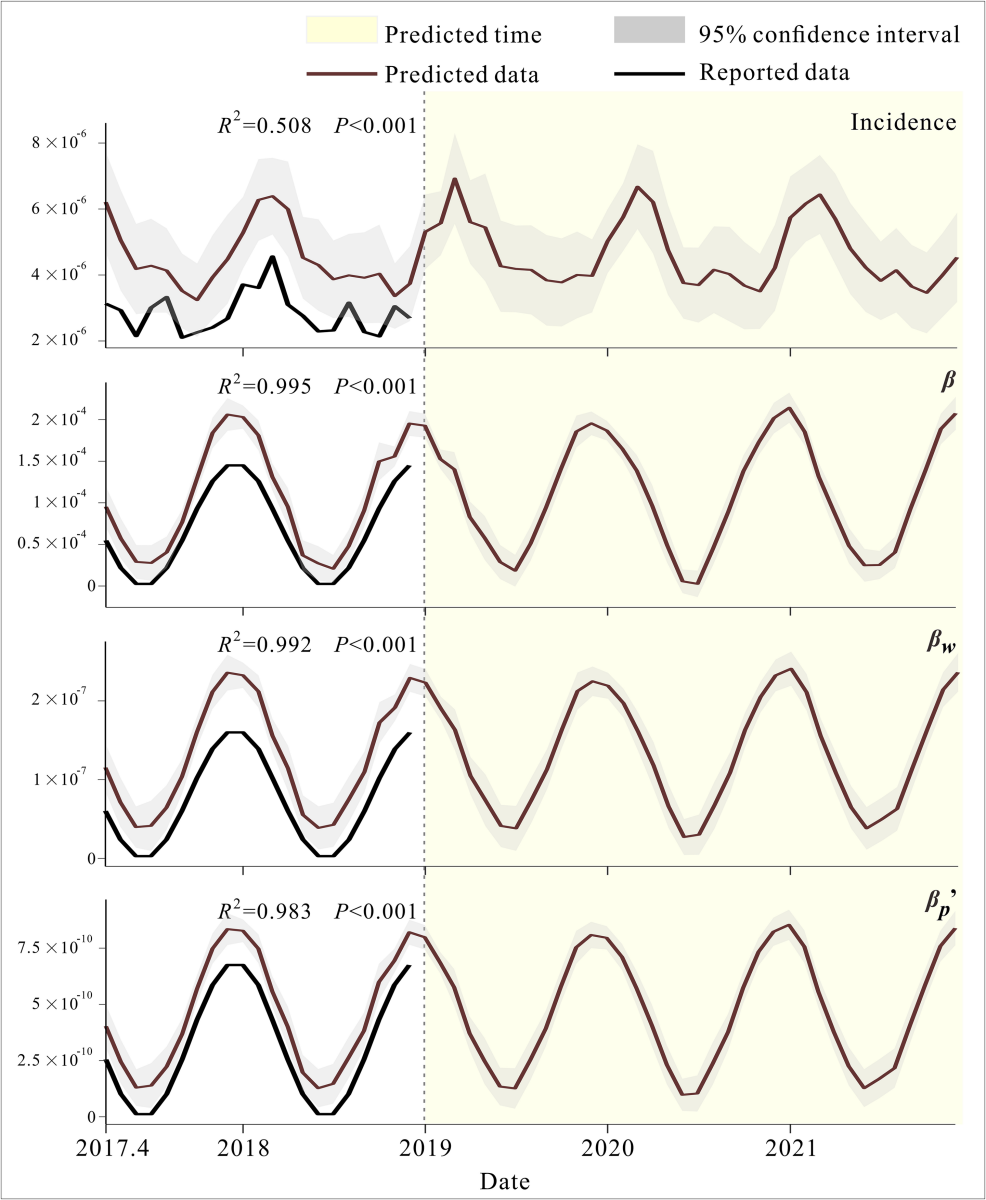
Validation and prediction of the GAM model.

We applied the GAM to predict changes in incidence rates and transmission rate coefficients across different pathways from April 2017 to December 2022 used for model validation. As shown in the figure, the fitted trends for incidence rates and transmission rate coefficients across different pathways were generally consistent, and the validation results for all four models were statistically significant (*p* < 0.001). GAM predictions for incidence and all three transmission rate coefficients exhibited clear seasonal patterns, with incidence showing a bimodal trend within a year (Figure 6).

## 4 Discussion

Hepatitis E is often regarded as an infectious disease primarily confined to regions with poor sanitation and contaminated drinking water supplies. However, as it is also a zoonotic disease with certain transmission routes still not fully understood, an increasing number of cases have been reported in non-endemic areas, including Jiangsu Province, China. Therefore, investigating the factors influencing hepatitis E transmission is particularly important.

Numerous studies have demonstrated that infectious diseases are sensitive to climate (27–29). In recent years, the impact of meteorological factors such as humidity, temperature, and precipitation on the epidemiology of hepatitis E has garnered significant attention (20, 30, 31). This study combined the Generalized Additive Model (GAM) with a transmission dynamics model to explore the relationships between meteorological factors and the incidence of hepatitis E, as well as the transmission rate coefficients (*β*, *β*_w_, and *β*_p_′) across three pathways. The results indicate that the influence of meteorological factors on three pathways followed similar trends. In the optimal model’s predictions for incidence, a clear bimodal trend was observed, suggesting that the seasonal dual-peak fluctuations in Jiangsu Province are primarily associated with average atmospheric pressure, sunshine duration, wind speed, and relative humidity (32). However, the validation results of the GAM model showed that the model’s performance in predicting incidence was relatively weaker compared to its fit for transmission rate coefficients. This discrepancy may be attributed to changes in data units or may indicate that meteorological factors influence incidence through mechanisms beyond discussed transmission.

Furthermore, precipitation showed a non-significant impact on hepatitis E incidence (*p* > 0.05), and its effect on the reservoir-to-person transmission rate coefficient remained marginal, contrasting with previous reports (24). Four potential reasons may explain this discrepancy: First, HEV genotypes 3/4 (dominant in China) exhibit weaker environmental linkages compared to waterborne genotypes 1/2 prevalent in developing countries (7, 33, 34). Second, as a socioeconomically advanced region, Jiangsu Province has minimized large-scale water contamination risks (35). Third, the MHMRTDM model may inadequately capture minor August incidence peaks coinciding with monsoon rainfall. Finally, unaddressed lag effects of precipitation could attenuate its observed influence.

In contrast, relative humidity significantly affected all dependent variables. Higher humidity consistently reduced transmission rate coefficients across pathways, likely by limiting human exposure opportunities. The steeper decline in host-to-person transmission above 75% humidity, which may be due to the fact that increased humidity and precipitation limit human production activities (36), reducing contact between humans and hosts.

The lack of a significant association between temperature and host-to-person transmission may result from opposing mechanisms operating at different ends of the temperature spectrum: higher temperatures may reduce HEV environmental viability and persistence (37), while lower temperatures may suppress human outdoor activity and inter-host contact (38). These counteracting effects could balance each other out across the observed temperature range, leading to a net null effect in the model. Peak transmission at 1013 hPa (near optimal human comfort pressure) suggests active host interactions under favorable conditions. Previous studies have shown that the host-to-person transmission rate coefficient contributes the most to the spread of hepatitis E. The results of this study indicate that *β*_p_′ is more sensitive to meteorological factors compared to the other two transmission pathways, highlighting the importance of focusing on meteorological factors for controlling host-to-human transmission, which is crucial for hepatitis E prevention and control.

This study has several limitations. As noted, the lack of consideration of lag effects stems from using monthly data for both the variables and the MHMRTDM model. Additionally, the omission of external factors may limit the model’s ability to replicate observed trends. Future research will address these issues by using higher-frequency data (e.g., daily or weekly) and incorporating external factors to improve predictive accuracy. The relationships identified by the GAM between climate variables and transmission dynamics are correlative and do not imply causality. Future studies should incorporate causal inference methods, such as instrumental variable approaches or mechanistic models, to further explore these associations.

## Data Availability

The data analyzed in this study is subject to the following licenses/restrictions: the incidence data of hepatitis E were provided by the Jiangsu Provincial Center for Disease Control and Prevention (CDC). Meteorological data were collected from the Jiangsu Provincial Meteorological Bureau. Although both data sources have undergone sensitive information scrubbing, they still remain unsuitable for public disclosure. The effort towards disease control is part of the CDC’s routine responsibility in Jiangsu Province, China. Therefore, institutional review and informed consent were not required for this study. All data analysed were anonymised. Requests to access these datasets should be directed to 2578123951@qq.com.

## Supplementary material

SUPPLEMENTARY FIGURE S1 Established the transmission dynamics of the MHMRTDM model of hepatitis E. S_i_, Susceptible individual density; E_i_, Exposed individual density; I_i_, Infectious individual density; A_i_, Asymptomatic individual density; R_i_, Recovered/removed individual density; W, Pathogen concentration in water reservoir; N_i_, Total population density; N_p_, Total host herd density; S_p_, Susceptible host density; E_p_, Exposed host density; I_p_, Infectious host density; D_p_, Slaughtered host density. Reprinted with permission from (18), licensed under CC BY 4.0.

SUPPLEMENTARY TABLE S1 Sensitivity analysis of *β* parameters to climate variables using Pearson correlation, linear regression, and GAM.

SUPPLEMENTARY FIGURE S2 Visualization of sensitivity analysis with GAM-fitted smooth curves.

## References

[1] Chen YJ Cao NX Xie RH Ding CX Chen EF Zhu HP. Epidemiological investigation of a tap water-mediated hepatitis E virus genotype 4 outbreak in Zhejiang Province, China. Epidemiol Infect. (2016) 144:3387–99. doi: 10.1017/S0950268816001898, PMID: 27546066 PMC9150197

[2] Lake IR. Food-borne disease and climate change in the United Kingdom. Environ Health. (2017) 16:117. doi: 10.1186/s12940-017-0327-0, PMID: 29219100 PMC5773878

[3] Wenjing YE Zhang C Chen C. Analysis of the association between intestinal infectious diseases and climate factors of Fujian Province in 2006—2015. J Med Theory Pract. (2018) 31:3333–3337. doi: 10.19381/j.issn.1001-7585.2018.22.007

[4] Chan MCW Kwok K Hung TN Chan PKS. Molecular epidemiology and strain comparison between hepatitis E viruses in human sera and pig livers during 2014 to 2016 in Hong Kong. J Clin Microbiol. (2017) 55:1408–15. doi: 10.1128/JCM.02020-16, PMID: 28202801 PMC5405258

[5] Miyamura T. Hepatitis E virus infection in developed countries. Virus Res. (2011) 161:40–6. doi: 10.1016/j.virusres.2011.03.006, PMID: 21443914

[6] Song YJ Jeong HJ Kim YJ Lee SW Lee JB Park SY. Analysis of complete genome sequences of swine hepatitis E virus and possible risk factors for transmission of HEV to humans in Korea. J Med Virol. (2010) 82:583–91. doi: 10.1002/jmv.21730, PMID: 20166181

[7] Yugo DM Meng XJ. Hepatitis E virus: foodborne, waterborne and zoonotic transmission. Int J Environ Res Public Health. (2013) 10:4507–33. doi: 10.3390/ijerph10104507, PMID: 24071919 PMC3823334

[8] Drabick JJ Gambel JM Gouvea VS Caudill JD Sun W Hoke CH Jr. A cluster of acute hepatitis E infection in United Nations Bangladeshi peacekeepers in Haiti. Am J Trop Med Hyg. (1997) 57:449–54. doi: 10.4269/ajtmh.1997.57.449, PMID: 9347962

[9] Labrique AB Thomas DL Stoszek SK Nelson KE. Hepatitis E: an emerging infectious disease. Epidemiol Rev. (1999) 21:162–79. doi: 10.1093/oxfordjournals.epirev.a017994, PMID: 10682255

[10] Isaacson M Frean J He J Seriwatana J Innis BL. An outbreak of hepatitis E in northern Namibia, 1983. Am J Trop Med Hyg. (2000) 62:619–25. doi: 10.4269/ajtmh.2000.62.619, PMID: 11289674

[11] Skidmore SJ. Factors in spread of hepatitis E. Lancet. (1999) 354:1049–50. doi: 10.1016/S0140-6736(99)00241-X, PMID: 10509491

[12] WHO. (2014). Waterbone outbreaks of hepatitis E: recognition, investigation and control: technical report. Available online at: https://www.who.int/publications/i/item/waterborne-outbreaks-of-hepatitis-e-recognition-investigation-and-control

[13] Piper-Jenks N Horowitz HW Schwartz E. Risk of hepatitis E infection to travelers. J Travel Med. (2000) 7:194–9. doi: 10.2310/7060.2000.00059, PMID: 11003732

[14] Ayouni K Naffeti B Ben Aribi W Bettaieb J Hammami W Ben Salah A. Hepatitis a virus infection in central-West Tunisia: an age structured model of transmission and vaccination impact. BMC Infect Dis. (2020) 20:627. doi: 10.1186/s12879-020-05318-7, PMID: 32842988 PMC7477833

[15] Bao KB Zhang QM Li XN. A model of Hbv infection with intervention strategies: dynamics analysis and numerical simulations. Math Biosci Eng. (2019) 16:2562–86. doi: 10.3934/mbe.2019129, PMID: 31137228

[16] Jayasundara D Hui BB Regan DG Heywood AE Macintyre CR Wood JG. Modelling the decline and future of hepatitis a transmission in Australia. J Viral Hepat. (2019) 26:199–207. doi: 10.1111/jvh.13018, PMID: 30315680

[17] Chen T He Q Tan A. Development of a multiple transmission pathways dynamic model of hepatitis E and its application dynamics in Changsha. Chin J Health Stat. (2014) 31:257–62.

[18] Yang M Cheng XQ Zhao ZY Li PH Rui J Lin SN. Feasibility of controlling hepatitis E in Jiangsu Province, China: a modelling study. Infect Dis Poverty. (2021) 10:91. doi: 10.1186/s40249-021-00873-w, PMID: 34187566 PMC8240442

[19] Chen XX Shi Y Lu YH Chen YH Chen KY Ren H. Spatial-temporal distribution characteristics of hepatitis E in Shanghai, 2006-2016. Zhonghua Liu Xing Bing Xue Za Zhi. (2018) 39:971–6. doi: 10.3760/cma.j.issn.0254-6450.2018.07.020, PMID: 30060314

[20] Guo Y Feng Y Qu F Zhang L Yan B Lv J. Prediction of hepatitis E using machine learning models. PLoS One. (2020) 15:e0237750. doi: 10.1371/journal.pone.0237750, PMID: 32941452 PMC7497991

[21] Liu Y Liang W Li J Liu F Zhou G Zha W. Characteristic of spatial-temporal distribution of hepatitis E in Hunan province, 2006-2014. Zhonghua Liu Xing Bing Xue Za Zhi. (2016) 37:543–7. doi: 10.3760/cma.j.issn.0254-6450.2016.04.021, PMID: 27087223

[22] Liu ZQ Zuo JL Yan Q Fang QW Zhang TJ. Epidemiologic and spatio-temporal characteristics of hepatitis E in China, 2004-2014. Zhonghua Liu Xing Bing Xue Za Zhi. (2017) 38:1380–5. doi: 10.3760/cma.j.issn.0254-6450.2017.10.017, PMID: 29060984

[23] Peng T Chen X Wan M Jin L Wang X Du X. The prediction of hepatitis E through ensemble learning. Int J Environ Res Public Health. (2020) 18:159. doi: 10.3390/ijerph18010159, PMID: 33379298 PMC7795791

[24] Tricou V Bouscaillou J Laghoe-Nguembe GL Bere A Konamna X Selekon B. Hepatitis E virus outbreak associated with rainfall in the Central African Republic in 2008-2009. BMC Infect Dis. (2020) 20:260. doi: 10.1186/s12879-020-04961-4, PMID: 32245368 PMC7119096

[25] Imai C Hashizume M. A systematic review of methodology: time series regression analysis for environmental factors and infectious diseases. Trop Med Health. (2015) 43:1–9. doi: 10.2149/tmh.2014-21, PMID: 25859149 PMC4361341

[26] Shackleton D Economou T Memon FA Chen A Dutta S Kanungo S. Seasonality of cholera in Kolkata and the influence of climate. BMC Infect Dis. (2023) 23:572. doi: 10.1186/s12879-023-08532-1, PMID: 37660078 PMC10474634

[27] Semenza JC Menne B. Climate change and infectious diseases in Europe. Lancet Infect Dis. (2009) 9:365–75. doi: 10.1016/S1473-3099(09)70104-5, PMID: 19467476

[28] Semenza JC Suk JE Estevez V Ebi KL Lindgren E. Mapping climate change vulnerabilities to infectious diseases in Europe. Environ Health Perspect. (2012) 120:385–92. doi: 10.1289/ehp.1103805, PMID: 22113877 PMC3295348

[29] Xiang J Hansen A Liu Q Liu X Tong MX Sun Y. Association between dengue fever incidence and meteorological factors in Guangzhou, China, 2005-2014. Environ Res. (2017) 153:17–26. doi: 10.1016/j.envres.2016.11.009, PMID: 27883970

[30] Johne R Wolff A Gadicherla AK Filter M Schluter O. Stability of hepatitis E virus at high hydrostatic pressure processing. Int J Food Microbiol. (2021) 339:109013. doi: 10.1016/j.ijfoodmicro.2020.109013, PMID: 33340943

[31] Ren H Li J Yuan ZA Hu JY Yu Y Lu YH. The development of a combined mathematical model to forecast the incidence of hepatitis E in Shanghai, China. BMC Infect Dis. (2013) 13:421. doi: 10.1186/1471-2334-13-421, PMID: 24010871 PMC3847129

[32] Carratala A Joost S. Population density and water balance influence the global occurrence of hepatitis E epidemics. Sci Rep. (2019) 9:10042. doi: 10.1038/s41598-019-46475-3, PMID: 31296895 PMC6624372

[33] Khuroo MS Khuroo MS Khuroo NS. Transmission of hepatitis E virus in developing countries. Viruses. (2016) 8:253. doi: 10.3390/v8090253, PMID: 27657112 PMC5035967

[34] Kmush BL Nelson KE Labrique AB. Risk factors for hepatitis E virus infection and disease. Expert Rev Anti-Infect Ther. (2015) 13:41–53. doi: 10.1586/14787210.2015.981158, PMID: 25399510

[35] WHO. (2010). The global prevalence of hepatitis E virus infection and susceptibility. Available online at: https://www.who.int/publications/i/item/Who-Ivb-10.14

[36] Wang J Tang K Feng K Lin X Lv W Chen K. Impact of temperature and relative humidity on the transmission of COVID-19: a modelling study in China and the United States. BMJ Open. (2021) 11:e043863. doi: 10.1136/bmjopen-2020-043863, PMID: 33597143 PMC7893211

[37] Emerson SU Arankalle VA Purcell RH. Thermal stability of hepatitis E virus. J Infect Dis. (2005) 192:930–3. doi: 10.1086/432488, PMID: 16088844

[38] Olczak K Nowicki J Klocek C. Pig behaviour in relation to weather conditions - a review. Ann Anim Sci. (2015) 15:601. doi: 10.1515/aoas-2015-0024

