# Supplementary figures and images for: Meteorological determinants of hepatitis E dynamics in Jiangsu Province, China: a pre-COVID-19 era study focusing on multi-route transmission (2005–2018)
Source: Front Public Health. 2025 Aug 7;13:1604579. doi: 10.3389/fpubh.2025.1604579 (PMC12367770; doi:10.3389/fpubh.2025.1604579)

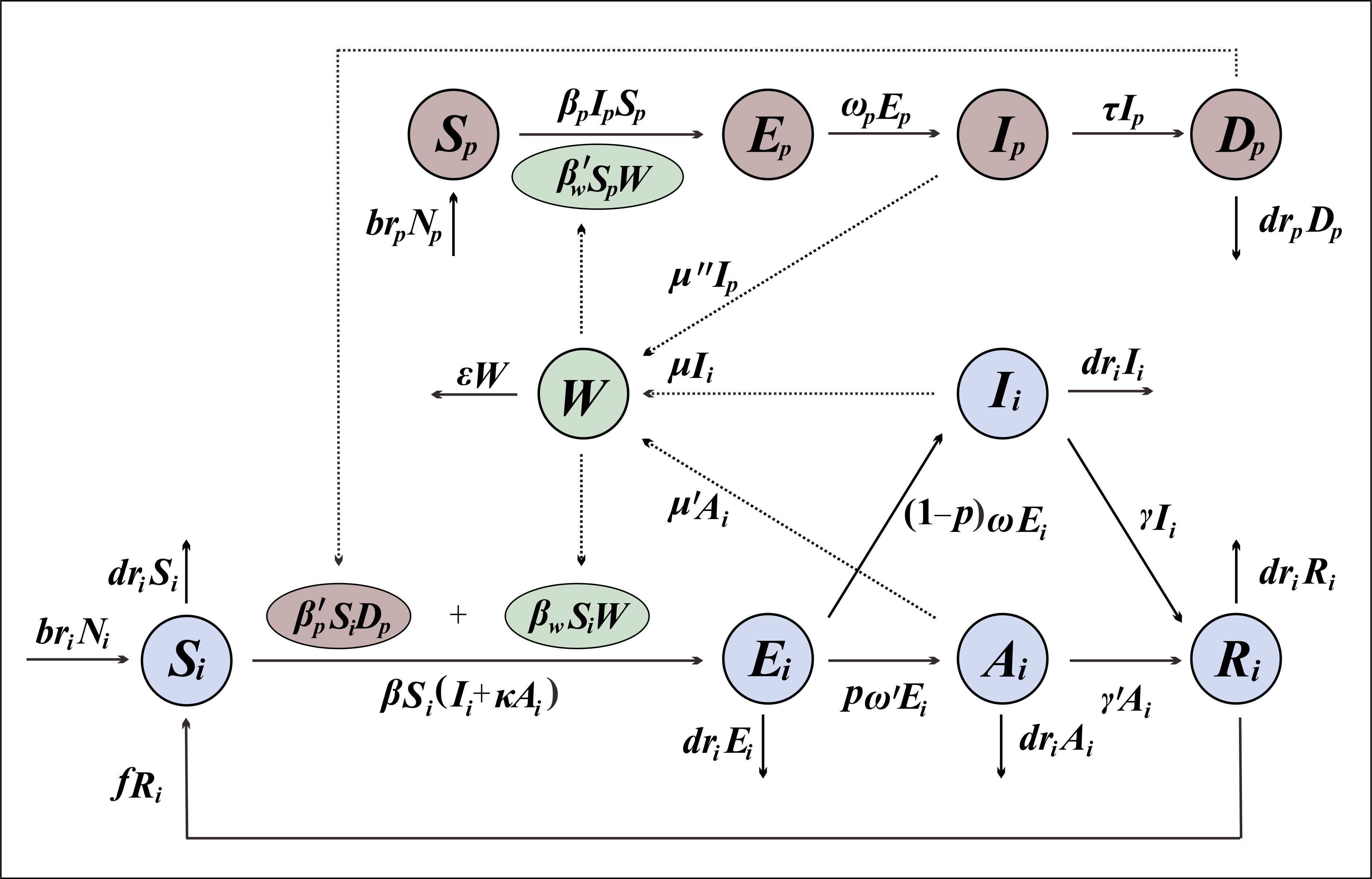

Supplement: SUPPLEMENTARY FIGURE S1 — Established the transmission dynamics of the MHMRTDM model of hepatitis E. Si, Susceptible individual density; Ei, Exposed individual density; Ii, Infectious individual density; Ai, Asymptomatic individual density; Ri, Recovered/removed individual density; W, Pathogen concentration in water reservoir; Ni, Total population density; Np, Total host herd density; Sp, Susceptible host density; Ep, Exposed host density; Ip, Infectious host density; Dp, Slaughtered host density. Reprinted with permission from (18), licensed under CC BY 4.0. [file Image_1.jpg]

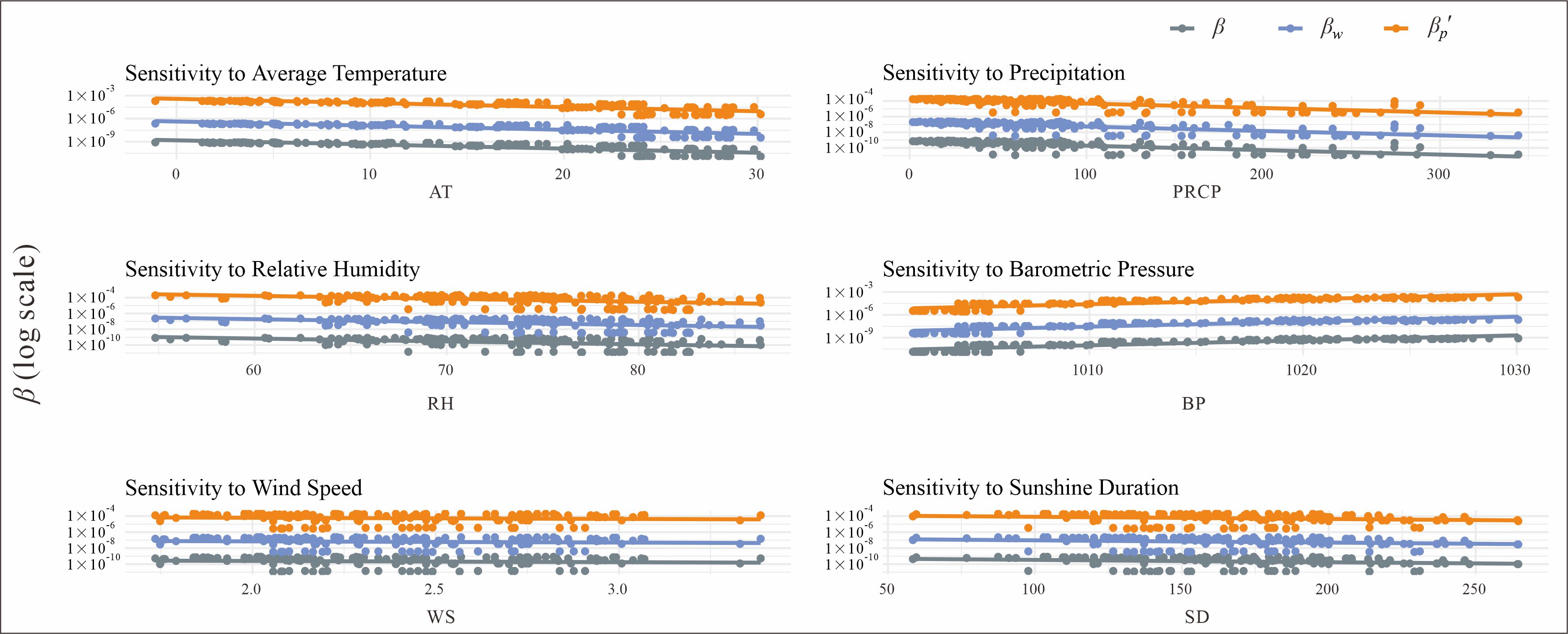

Supplement: SUPPLEMENTARY FIGURE S2 — Visualization of sensitivity analysis with GAM-fitted smooth curves. [file Image_2.jpeg]
