# Supplementary material for: Meteorological determinants of hepatitis E dynamics in Jiangsu Province, China: a pre-COVID-19 era study focusing on multi-route transmission (2005–2018)
Source: Front Public Health. 2025 Aug 7;13:1604579. doi: 10.3389/fpubh.2025.1604579 (PMC12367770; doi:10.3389/fpubh.2025.1604579)
Supplement: SUPPLEMENTARY TABLE S1 — Sensitivity analysis of β parameters to climate variables using Pearson correlation, linear regression, and GAM. [file Table_1.docx]

Correlation with Climate Variables

| Variable | BP | SD | RH | AT | PRCP | WS |
| --- | --- | --- | --- | --- | --- | --- |
| *β* | 0.9491314 | -0.3668006 | -0.4156152 | -0.9078875 | -0.6789005 | -0.1471800 |
| *β*_w_ | 0.9568503 | -0.3791904 | -0.4123062 | -0.9109939 | -0.6796620 | -0.1411315 |
| *β*_p_′ | 0.9606142 | -0.3871079 | -0.4046014 | -0.9133772 | -0.6759903 | -0.1449289 |

Regression Slopes with Climate Variables

| Variable | BP | SD | RH | AT | PRCP | WS |
| --- | --- | --- | --- | --- | --- | --- |
| *β* | 7.91E-06 | -6.20E-07 | -4.28E-06 | -6.98E-06 | -6.13E-07 | -2.81E-05 |
| *β*_w_ | 8.94E-09 | -7.19E-10 | -4.76E-09 | -7.86E-09 | -6.89E-10 | -3.03E-08 |
| *β*_p_′ | 3.20E-11 | -2.62E-12 | -1.67E-11 | -2.81E-11 | -2.44E-12 | -1.11E-10 |
